# Evolution of antiviral host defenses against a backdrop of endogenous retroelements

**DOI:** 10.1126/science.adx4379

**Published:** 2025-08-07

**Authors:** George Kassiotis, Jonathan P. Stoye

**Affiliations:** 1 The Francis Crick Institute, London, UK; 2 Department of Infectious Disease, Faculty of Medicine, Imperial College London, London, UK

## Abstract

Mammalian hosts deploy a multitude of germline-encoded mechanisms to detect and restrict virus infection. These must avoid pathological responses to endogenous retroviruses (ERVs) and other endogenous retrotransposable elements (RTEs) – viruses and virus-like genomic parasites that have invaded the host germline and are passed down the generations as host genes. Although the location, specificity and sensitivity of innate pattern-recognition receptors (PRRs) and restriction factors are tuned to facilitate discrimination of infecting viruses from those that are part of self, immune cross-reactions do occur. The RTE viral heritage may therefore compromise the ability of the host to respond to virus infection without risking pathology. Nevertheless, it also provides opportunities for RTEs to be co-opted as an alarm amplification system, repurposed as antiviral factors and contribute to the evolution of antiviral genes.

## Introduction

The evolutionary success of an organism is determined by replication of its genome in the face of exposure to a wide range of infectious parasites. All organisms have evolved defenses against infection (1). In mammals, distinct germline-encoded defenses cell-autonomously sense and neutralize invading viruses, and trigger signaling cascades alerting neighboring cells and the immune system to the presence of the invader. These defenses target evolutionarily conserved structures, such as viral capsids, which protect the invading genomes, viral envelope glycoproteins, which attach and fuse virus particles with the target cells, and viral genomes and their replication intermediates, all of which present patterns distinct from the host.

Restriction factors are germline-encoded host defense molecules that can directly limit virus replication by interfering with one or more steps in virus replication or by restricting the availability of cellular resources. Pattern-recognition receptors (PRRs) can sense virus-specific patterns and initiate a signaling cascade, leading to the production of interferons (IFNs) and induction of IFN-stimulated genes (ISGs) with antiviral activity. Although some restriction factors and PRRs are expressed constitutively, many are IFN-inducible, bolstering antiviral defenses following virus sensing. Moreover, certain restriction factors may either directly initiate pro-inflammatory signaling cascades or indirectly facilitate sensing by dedicated PRRs, connecting the network of innate antiviral defenses.

However, these defenses cannot evolve to provide complete resistance – otherwise, there would be no selection pressure to maintain them in the absence of exposure (1). Rather they provide sufficient resistance to ensure the survival of the host, so both host and virus species can survive. Moreover, these mechanisms can be potentially as damaging for the host as for the virus, and their deployment must be specific and proportionate to the risk of infection. During the ensuing arms race, viruses evolve countermeasures that allow them to evade or subvert host defenses and establish acute or chronic infection in somatic cells.

**Figure 1:**
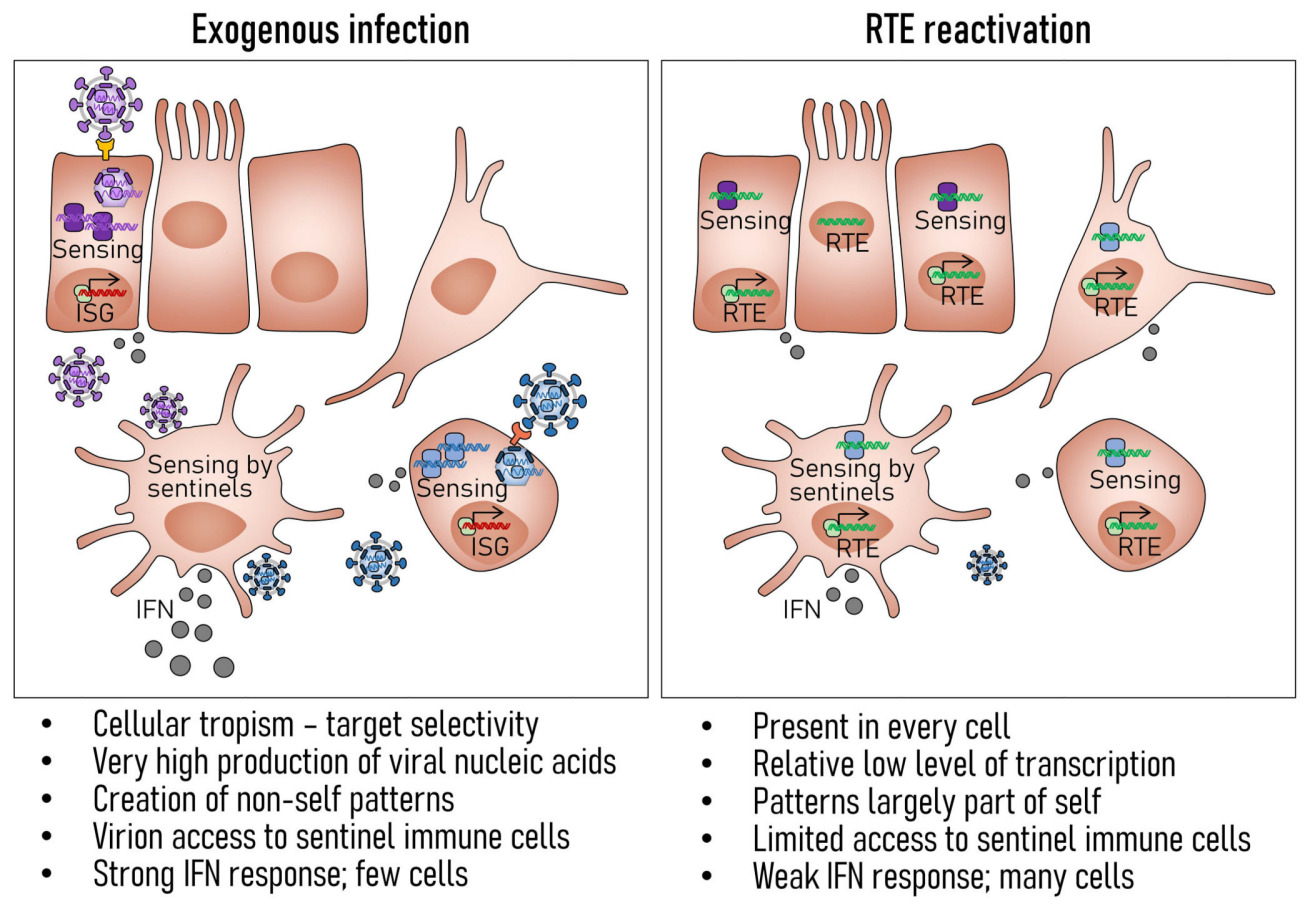
Distinct immunogenic features of exogenous viruses and RTEs. Exogenous viruses typically target one or few cell types, where they produce excessive amounts of viral nucleic acids, often with distinctive molecular patterns and recognized by common or cell type-specific sensors. Their virions also access specialized sentinel cells, such as pDCs, which become the predominant sources of IFN. In contrast, RTEs are present and can be reactivated in every nucleated cell, but are rarely transcribed at high levels, or produce specific molecular patterns and extracellular particles that would access pDCs.

The situation becomes more complicated when we consider retroviruses, viruses that must integrate into host DNA as part of their replication cycle. Some can invade the host germline, thus becoming endogenous and blurring the boundaries between host and virus. These elements, termed endogenous retroviruses (ERVs), join other types of retrotransposable elements (RTEs) in the genome, with which they share a common ancestry (2) and the ability to amplify their DNA copies in the host germline through the reverse transcription and reintegration of their RNA transcripts into DNA. There are now over 4 million RTE integrations in the human genome – making up ˜42% of our DNA – which belong to distinguishable groups (2, 3), with distinct replication life-cycles and with specific consequences for the host innate and adaptive immune systems (Figure 1).

Through accumulated mutations and deletions, the vast majority of RTEs have lost the ability to replicate or retrotranspose. In addition, they are suppressed by epigenetic mechanisms developed in response to these genomic parasites. Nevertheless, some RTEs are still able to produce replication intermediates and proteins that can activate antiviral immunity. This creates a challenge for the host to discriminate between the few RTEs that retain pathogenic potential from those that no longer pose a threat or have even been co-opted in host physiology, whilst still maintaining sufficient responsiveness to exogenous virus infection. Here, we review how host antiviral defenses have evolved in the context of RTEs, and discuss the surprising opportunities and ammunition against other infections that RTEs provide for their host.

### Guarding against cellular entry and exit of viruses

Perhaps most effective defenses against infection are barriers to the ingress of the invader’s genetic material through the membrane of the host cell. Entry of enveloped viruses requires attachment to cellular receptors and fusion of lipid membranes. Several host factors have evolved to block this step or reduce its efficiency. Where this defense succeeds, no further host action may be necessary. Processes required for virus entry are, therefore, frequently targeted by host restriction factors.

A recurring mechanism in host defense is the expression of intact or mutated envelope glycoproteins from ERVs, which blocks entry of exogenous viruses in a manner resembling viral superinfection resistance, whereby infection with a virus that uses a particular cellular entry receptor prevents entry of other viruses that use the same receptor. This has led to the convergence in evolution of exogenous retrovirus restriction in many species, including mice, cats and sheep (Table 1) (4). A preserved copy of the human endogenous retrovirus (HERV)-T envelope glycoprotein that can bind its cognate receptor, MCT-1, without causing membrane fusion, may have led to the extinction of this family of exogenous retroviruses in our primate ancestors (5). Syncytin-1, derived from a HERV-W envelope glycoprotein and co-opted in human placentation, as well as the phylogenetically distant ERVH48-1 envelope glycoprotein (also known as Suppressyn) bind to ASCT2 as a receptor. ASCT2 acts as a cellular receptor for several other retroviruses, including currently circulating primate retroviruses, and Syncytin-1 and ERVH48-1 binding to ASCT2 can, consequently, inhibit infection with diverse endogenous and exogenous retroviruses (6, 7).

Efficient transmission of exogenous mouse mammary tumor viruses (MMTVs) relies on induction of T cell proliferation by virally-encoded superantigens. Endogenous MMTV proviruses promote thymic deletion of superantigen-reactive T cells (3), thus providing relative resistance to exogenous MMTV infection through a unique mechanism.

Viral egress also represents an important target for virus restriction where ERV products may play a role. ERV envelope glycoproteins can inhibit retroviruses that use unrelated receptors. For example, defective envelope glycoproteins from the most recently endogenized HERV-K(HML-2) family have been shown to limit production of human immunodeficiency virus-1 (HIV-1) in an incompletely understood manner (8). Tetherin (encoded by *BST2*) is a restriction factor that prevents the release of enveloped virus particles from infected cells by physically tethering them to the plasma membrane of the producer cell. It is active against most tested viruses, including HERV-K(HML-2). In addition to this direct activity, virus assembly at the plasma membrane can be sensed as a molecular pattern by Tetherin, triggering NF-κB-dependent proinflammatory responses, and Tetherin-mediated accumulation of virus particles may also facilitate sensing by other receptors (9).

Several exogenous viruses can inactivate Tetherin, albeit using different mechanisms. Such antagonistic activity seems to be retained by the envelope glycoprotein of some HERV-K(HML-2) proviruses, expression of which at the plasma membrane can inactivate Tetherin (10), potentially facilitating instead of inhibiting exogenous virus release. As HERV-K(HML-2) envelope glycoproteins are variably expressed between individuals and certain proviruses are insertionally polymorphic – present in some individuals but not others – they may differentially affect susceptibility to exogenous infection by disparate effects on Tetherin (10).

The apparent success of restriction mechanisms blocking viral entry and exit may be responsible for the evolution of an RTE life cycle involving intracellular genome amplification without the need for cell-to-cell transmission or somatic cell infection (11), further complicating the host’s task in distinguishing endogenous from exogenous virus replication (3).

### Targeting incoming viral genomes

If blocks at the cell membrane are circumvented, a second tier of defenses awaits. These include restriction factors that act to prevent the synthesis of complete complementary DNA (cDNA) copies of the viral genome or its access to the host genome for integration (12). A key target is the viral capsid protein that forms a shell enclosing the viral replication machinery and preventing sensing of viral nucleic acids. Examples of restriction factors that target the viral capsid include TRIM5α, a member of the large family of proteins bearing the Tripartite Motif, many of which contribute to antiviral immunity, and Fv1 which is derived from a murine endogenous retrovirus L family (MuERV-L) RTE and has followed an evolutionary pathway spawning variants with specific recognition properties for different retroviruses (13). TRIM5α appears to act by causing premature uncoating of the viral core inhibiting cDNA synthesis or exposing the viral nucleic acids to TREX1, which digests cytosolic DNA and cDNA, while Fv1 most likely prevents core transit to the nucleus (14).

As with antiviral restriction factors targeting envelope glycoprotein-mediated virus entry and exit, those targeting the viral capsid can also act at multiple steps of the viral life-cycle. For example, the Gag protein from an endogenous HERV-K(HML-2) provirus can poison HIV-1 core assembly (15), as does the Gag protein of endogenous Jaagsiekte sheep retrovirus (JSRV), which inhibits assembly of the cognate infectious retrovirus (16). These examples further underscore the repeated co-option of ERV proteins as restriction factors that provide genetic resistance to retroviral infection.

### Triggering an IFN response

Successful entry of a virus particle into a cell warrants a response, proportional to the extent of subsequent virus replication. This requires fast and accurate detection of the invader, coupled to immediate block of replication in the infected cell, as well as of virus spread to other cells. Detection is achieved by a battery of strategically placed PRRs, including Toll-like receptors (TLRs), RIG-I-like receptors (RLRs), NOD-like Receptors (NLRs) and AIM2-like receptors (ALRs), that recognize diverse pathogen-associated molecular patterns (PAMPs), but may also bind ligands produced by the host cell. Several mechanisms have been described that maximize the ability of PRRs to sense infection, whilst limiting reactivity with self-generated ligands, including those from RTEs (17).

PRRs can evolve specificity for structures that are produced exclusively or primarily during virus replication. They may also be expressed in a cell-specific manner or predominantly in specialized sentinels, such as plasmacytoid dendritic cells (pDCs) (Figure 1). Nevertheless, discrimination between self- and virus-produced PRR ligands is not always possible, necessitating additional mechanism of regulation.

Owing to 5’ capping and other modifications, Pol II-transcribed, host mRNAs are not sensed by RIG-I, which recognizes blunt-ended, double-stranded RNA (dsRNA), unmethylated at the 2’-O position, and with 5’ di- or tri-phosphates. However, Pol III-transcribed host RNAs, including 5S rRNA, and RTE RNAs, including the non-autonomous short interspersed nuclear elements (SINE), lack 5’ capping and, instead, avoid sensing by RIG-I by forming complex secondary structures or by interacting with RNA-binding proteins which shield them from detection (18). The dsRNA sensor MDA5, encoded by *IFIH1*, recognizes dsRNA longer than 300 base pairs (bp) produced during replication of certain viruses, as well as by the host cell. Ancestrally derived from the 7SL RNA gene, the primate-specific Alu subfamily of SINE have accumulated to over 1 million copies in the human genome, thus increasing the probability that two or more Alu repeats may be present in the same RNA transcript. Inverted Alu repeats that are sufficiently similar and proximal can form intramolecular RNA hairpins for the entire length of the Alu sequence, which averages ˜280 bp. This length, together with imperfect complementarity of the dsRNA region, further disrupted by ADAR-mediated editing, considerably reduces the ability of such hairpins to activate MDA5, suggesting the length of Alu sequence and tolerance of MDA5 to dsRNA mismatches are tuned to avoid this interaction (19). This balance is disrupted by gain-of-function *IFIH1* mutations or loss of ADAR activity, triggering spontaneous IFN production in immune disorders, including Aicardi-Goutières syndrome (AGS) and systemic lupus erythematosus (SLE) (20). Additional mechanisms that limit the availability of endogenous ligands for PRRs include nucleoside or nucleotide metabolizing enzymes such as SAMHD1, TREX1 and HELZ2, which digests highly structured RNAs (21).

When endogenous ligands cannot be efficiently modified or removed, their sensing can still be limited by regulated PRR expression and localization. Endosomal localization of the ligand-biding domains of dsRNA sensor TLR3, single-stranded RNA (ssRNA) sensors TLR7 and TLR8, and unmethylated CpG DNA sensor TLR9, also prevents detection of cytosolic ligands, and differences in intracellular localization also determine the targeting of host or virus RNA by ZAP (22). Although several PRRs are constitutively expressed in anticipation of infection, several require IFN to induce their transcription or a different isoform of the protein is induced following an IFN response. For example, in response to IFN, the *ADAR* gene produces a larger isoform that, in addition to dsRNA-binding domains, contains a domain (Zα) binding Z-form DNA or RNA. IFN signaling is a prerequisite for expression of the only other known mammalian protein with Zα domains, ZBP1, which can sense Z-form DNA or RNA, leading to further IFN induction or necroptosis.

Certain PRRs, including those that are inducible by IFN, can regulate the IFN response at the resolution phase of an infection. The *ZC3HAV1* gene encodes two isoforms of the RNA-binding protein ZAP that differ in length. While the longer isoform is constitutively expressed and targets viral RNA, the expression of the shorter isoform requires IFN and targets the mRNA of *IFN* genes for degradation, thereby negatively regulating the IFN response (22). Therefore, the IFN response to exogenous virus infection appears to be finely tuned by coordinated regulation of sensing and signaling cascades, as well as by negative feedback loops.

### Innate sensing of RTEs

An evolutionary trade-off between prevention of overt PRR engagement by RTEs and the necessity to maintain sensitivity to exogenous virus implies that RTE sensing may not be entirely avoided, particularly when homeostasis is disrupted. Moreover, as not all RTEs have been fully disarmed through evolution, the host must be prepared to sense their dysregulation to prevent further spread in the germline, as well as in somatic cells. Failure of such mechanisms is indicated by the frequent resurrection of ERVs in sensor- and immune-deficient mice (23, 24).

At least three conditions contribute to RTE products being expressed and sensed by cells. Firstly, epigenetic repression, which otherwise prevents RTE transcription, can be reversed during cellular infection, damage or malignant transformation, leading to the production of excessive RTE nucleic acids and proteins. Indeed, several exogenous viruses can upregulate RTE transcription, either through direct transactivation (25) or indirectly, in the case of SINE, through elevated transcription by host Pol III (18). Exogenous viruses also induce pro-inflammatory mediators, and target RTE repressors, restriction and other antiviral factors (26), all of which enhance RTE transcription indirectly. Infection with exogenous retroviruses in particular, can complement replication defects in ERVs, leading to their mobilization. Secondly, IFN production during viral infection or other pathologies, will additionally upregulate PRR expression which may facilitate detection of RTE products. Thirdly, to complete their own replication, viruses may also counteract restriction factors and sensor regulators, such as SAMHD1, Tetherin, PKR, and the APOBEC3 family, further enhancing the availability of RTE products for sensing.

**Figure 2:**
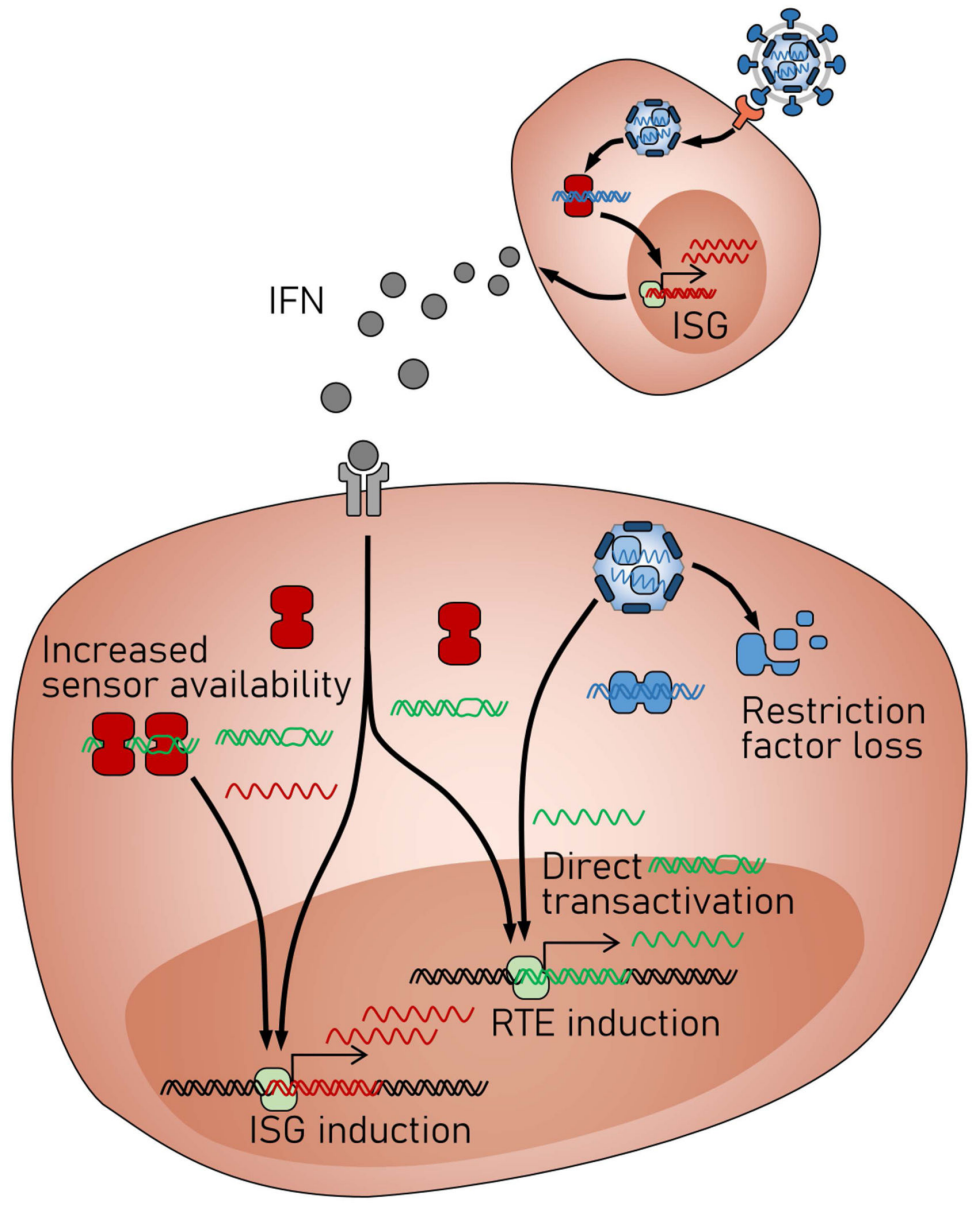
RTE activation and sensing triggered by viral infection. Infection with exogenous viruses increases RTE transcription and availability either directly, through transactivation by viral transcription factors, and indirectly thought pro-inflammatory mediators or by inactivating repressors restriction and other antiviral factors that normally silence or control RTEs. In parallel, IFN production during viral infection upregulates PRR expression. The combination of increased RTE transcription and reduced control, and increased PRR availability can initiate innate immune responses to RTEs, which, in turn, amplify responses to the exogenous infection.

The increased availability of RTE-derived ligands, coupled with increased PRR expression and reduced antiviral factor expression now permits the sensing of RTEs, which can contribute to the overall antiviral response of the host (Figure 2). Sensing of RTEs in the absence of infection or malignant transformation can directly cause or increase the risk of age-related inflammation, systemic autoimmunity or severe interferonopathies (3). However, it may be less pathogenic during virus infection, which should independently trigger an antiviral response. While certain viruses can benefit from the RTEs they induce (27), the contribution of induced RTEs to the antiviral response can enhance resistance to exogenous virus infection (26, 28).

### RTEs as regulators of adjacent innate immune gene function

Owing to their mutagenic potential and the regulatory sequences they carry, RTEs are a major force of host genome innovation. RTE integrations near or within genes can modify gene function on longer evolutionary timescales and with more profound consequences, and innate immune genes involved in sensing or restriction are no exception. RTE regulatory effects on genes that, in turn, affect resistance to infection or control RTE activity may have strong evolutionary effects.

For example, an intronic integration of a murine leukemia virus (MLV) long terminal repeat (LTR) in murine *Apobec3* enhances its expression and, consequently, its antiviral activity, thereby providing resistance to exogenous MLV infection (29). In human embryonic stem cells (ESCs), a HERV-H integration has been co-opted as a functional enhancer of *APOBEC3G*, and genome-wide HERV-H transcriptional activity is positively correlated with expression of the entire *APOBEC3* locus, as well as of the restriction factor *IFITM1* (30). A subfamily of murine B2 SINE repeats was found to contain STAT1-binding sites and function as IFN-inducible enhancers (31), and MER41_BT and Bov-A2 repeats play a similar role in bovine cells (32). Human MER41 repeats provide IFN inducibility to several antiviral sensors and effectors (33), including *AIM2*, a cytosolic dsDNA sensor, and *IFI6*, which exhibits broad antiviral activity (34, 35), but may also prevent RIG-I activation through competition for RNA ligands (36). Solitary *LTR12C* – single copies of the ERV9 subfamily LTR – also regulate expression and IFN inducibility of the downstream genes *GBP2* and *GBP5*, which inhibit envelope glycoproteins of exogenous viruses, as well as of ERVs, such as HERV-K(HML-2), being processed by the cellular protease furin (37, 38). An intronic *L1M2a* element has been co-opted as an enhancer of human *IFNAR1*, regulating both constitutive and IFN-inducible expression in B cells (39). Genetic loss of *TRIM28* in murine dendritic cells leads to the induction of proinflammatory and antiviral genes, including *Ifitm1* and *Gbp2b*, which has been attributed to regulatory control by adjacent derepressed RTEs (40). A copy of the Lx9c11 murine long interspersed nuclear element (LINE) subfamily has created the non-coding RNA *Lx9c11-RegoS*, which is indispensable in preventing lethal hyperinflammation during virus infection, through regulation of the genes encoding the Schlafen (SLFN) family of proteins (41).

Their cis-regulatory potential allows RTE integrations that are fixed in the genome to impact immune gene function negatively, too. Gene-dysregulating effects of RTEs are normally minimized by their epigenetic repression and suppression of their exonisation, but are particularly pronounced following malignant transformation, which is associated with RTE derepression, and may also manifest in other contexts. Transcriptional activation of a HERV-H provirus integrated downstream of the tandem *TLR7* and *TLR8* loci on human chromosome X initiates antisense transcription that is associated with loss of *TLR7* and *TLR8* transcription in several cancer types (42). Similarly, antisense transcription driven by a MER11C integration downstream of human *APOBEC3B* reduces its expression, without affecting expression of other APOBEC3 members on chromosome 22 in tumors of the male germline (42). Moreover, hypoxia-driven transcriptional activation of an intronic HERV-E provirus reduces the expression of *RNGTT* (42), encoding an enzyme that initiates 5’ capping of newly synthesized mRNAs, a modification essential for mRNA stability, translation and prevention of RIG-I sensing.

The cis-regulatory potential of germline RTEs may also modify the function of proteins involved in viral infection and concomitant innate immune response. ACE2 is a major entry receptor for seasonal and pandemic human coronaviruses, HCoV-NL63, SARS-CoV and SARS-CoV-2; and higher-affinity ACE2 binding is a critical but readily acquired trait for animal coronavirus spillover to humans (43). Evolutionary ancient intronic integrations of *MIRb* and *LTR16A1* repeats act as the promoter of an IFN-inducible, truncated *ACE2* isoform, thus coupling the IFN response to the induction of an ACE2 protein form that cannot mediate virus entry (44). A primate-specific, intronic *AluJr* repeat has been co-opted as an alternative terminal exon of *IFNAR2*, creating non-functional decoy receptor for type I IFNs that lacks the intracellular domain responsible for downstream signaling, thereby regulating the antiviral response (45). These examples highlight the immense potential of RTEs to alter or tune the function and expression of innate immune genes, with direct consequences for resistance to viral infection.

### Retention of viral heritage hinders co-option or causes pathology

Although RTEs are now part of the host genetic constitution and can be co-opted in essential host processes, this peaceful coexistence can sometimes be disrupted, causing such processes to fail.

The fusogenic potential of ERV envelope glycoproteins has been recurrently co-opted by placental mammals, in the form of the Syncytin genes, and function during the formation of the syncytiotrophoblast, a multinucleated layer of cells covering the placenta (46). Syncytin-mediated fusion of trophoblast cells follows the same principles as fusion during virus entry and may be subject to restriction by the same factors that have evolved to prevent the latter. Indeed, syncytiotrophoblast formation can be adversely affected by IFITMs (47). These are IFN-inducible restriction factors that inhibit the entry of several viruses by targeting envelope glycoproteins, and IFITM1 may also suppress several RTE families in human embryonic stem cells (48). Excessive activity of IFITMs induced by IFN or congenital infection can target Syncytins, by virtue of their retroviral origin, ultimately leading to complications in pregnancy (47). Furthermore, the antiviral activity of GBP5 can also inhibit the processing and fusogenic function of Syncytin-1 (49). These findings highlight a balance between the need to control potential pathogenic RTEs or exogenous viruses and the exapted function of Syncytins at that stage of human embryo development.

The promoter and enhancer function of HERV LTRs that has been co-opted in the transcription and IFN inducibility of innate immune genes represents another example where host mechanisms evolved to limit infection may modulate this alternative function. RTEs are epigenetically repressed, in part by a multi-gene family of KZNPs, which recruit the repressor TRIM28 (also known as KAP1) (50). Together with specialized mechanisms, such as PIWI-interacting RNAs (piRNAs), silencing RTEs specifically in the germline (51), KZNP-mediated repression may reduce the immediate risk to adjacent genes posed by cis-regulatory activity of newly-acquired RTEs, which can then be tested over longer evolutionary time for potentially beneficial activities through gradual or somatic cell type-specific release from KZNP control. However, excessive or persistent KZNP-mediated repression can restrict the evolutionary advantage that such cis-regulatory activity may provide. Indeed, reduced KZNP activity in subsets of elite controllers of HIV-1 infection has been correlated with increased LTR-driven expression of key antiviral genes, including *GBP2* and *GBP5* (52), suggesting that KZNP targeting of proviral LTRs may modulate the ability of exapted cis-regulatory LTRs to drive or otherwise regulate antiviral genes.

## Conclusions and future directions

Although the interaction of RTEs with host innate immunity may be pathogenic when dysregulated, or hinder the co-option of RTEs in physiological processes of the host, it also provides opportunities for the evolution of novel immune function. The current evidence supports a model, whereby the transcriptional responsiveness of RTEs to viral infection or malignant transformation may provide an alarm amplification system for such types of danger. Exogenous viruses can outpace host innate immunity with faster evolution of countermeasures. In contrast, RTE integrations follow the much slower evolutionary pace of the host genome. This increased genetic stability, together with their presence in every host cell, creates an ideal source of innate immune stimulation that can augment the immunogenicity of infected or transformed cells.

**Figure 3:**
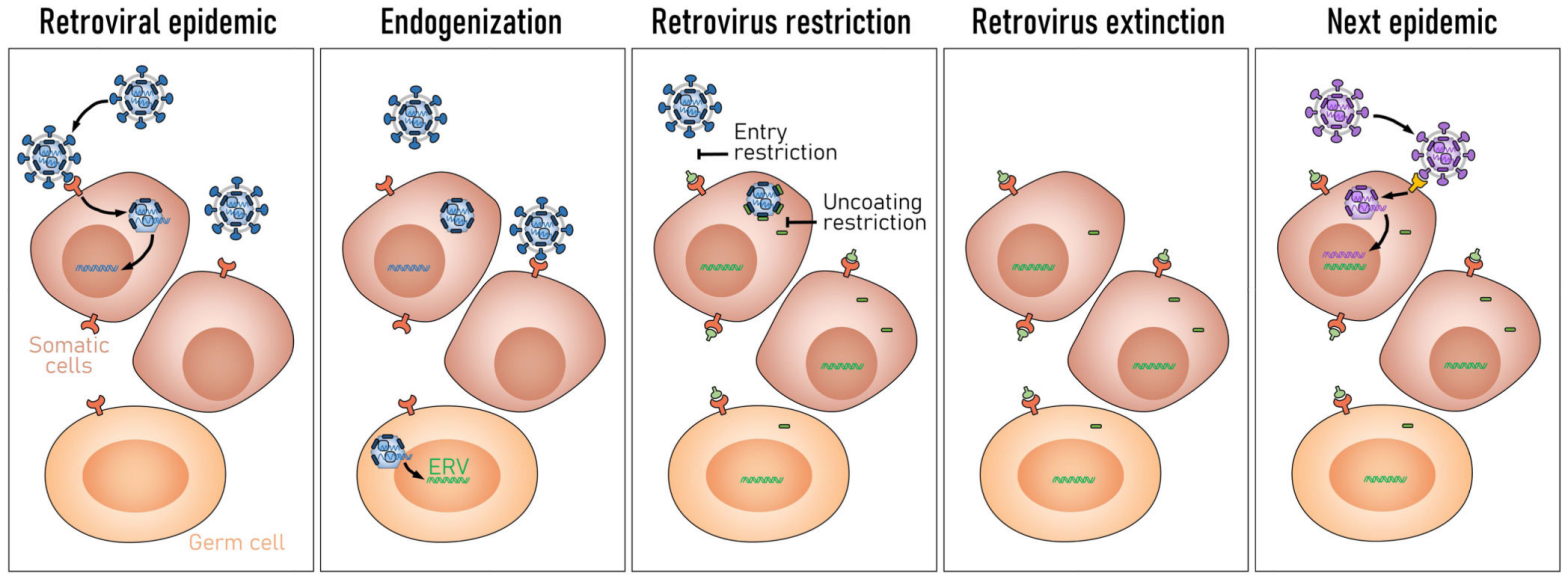
A proposed cycle of retroviral epidemic and evolution of innate resistance. Infection with a new exogenous retrovirus may occasionally cause germline infection leading to endogenization of one or more proviral copies (ERV). With long evolutionary time, ERVs are co-opted to produce defective products that interfere and restrict infection with related retroviruses, and achieve fixation in the host genome. When such restriction is efficient, it drives related exogenous viruses to extinction, but may not necessarily protect against more distant exogenous retroviruses, which can cause the next epidemic.

Inactivation of RTEs, either during the replication process or over evolutionary time also offers the opportunity to repurpose their individual components in the antiviral response, as exemplified by the utilization of defective ERV envelope glycoproteins and capsids as restriction factors. This creates a cycle, initiated by widespread infection with an exogenous retrovirus, occasionally leading to its endogenization and eventual inactivation of endogenous copies through mutation (Figure 3). Expression of defective components from the now endogenous copies may block infection with the cognate circulating retrovirus, ultimately leading to its extinction, and endogenous copies become fixed in the host germline (Figure 3). While the antiviral activities of defective ERVs might have been effective against ancient viral epidemics caused by their exogenous counterparts, they do not necessarily protect against modern-day or future viruses (53). The introduction of a retrovirus that bypasses existing ERV-derived antiviral activities initiates another cycle, until effective antiviral activities evolve further or are acquired de novo by the host.

In addition to their ability to trigger an IFN response and to the co-option of their components as restriction factors, genomic RTE integrations that are eventually allowed by the host to function as regulatory sequences contribute to genomic innovation, including the control of innate immunity genes. Identification of such functions may not be trivial. RTEs in the genome can be recognized by homology with consensus sequences. However, high sequence similarity between copies of the same RTE family within the same genome, as well as sequence and insertional polymorphisms between genomes complicate their analyses. Even more challenging is the annotation of transcripts that utilize or overlap with RTEs, particularly transcript isoforms that may characterize pathological conditions, such as malignant transformation (54, 55), although it will be facilitated by more comprehensive annotation of the diversity and distribution of RTEs in the human reference genome (56), and by advances in long-read RNA sequencing methodologies. The IFN response is also associated with characteristic shift in the transcriptome (57), and it would be important to characterize the contribution of RTEs in the creation of the IFN-inducible transcript isoforms. A potential contribution of RTEs is further supported by the association of their epigenetic state with inter-individual variation in the innate immune response to HIV-1 (52) and Influenza A (58). More comprehensive genomic and transcriptomic analyses of RTEs, including their inter-individual variation, will be an essential step toward our understanding of the evolutionary constraints our innate immune system faces in its fight against current and future exogenous viruses.

## Figures and Tables

**Table 1:** RTE-related antiviral sensors and restriction factors. Only mammalian antiviral sensors and restriction factors that are directly derived or shaped by RTEs are included. The species in which they were first described is also indicated. eMLV, ecotropic MLV; enJSRV, endogenous JSRV; ERV-DC, feline endogenous gammaretrovirus of the domestic cat; FeLV, Feline leukemia virus; MCF, mink cell focus-forming; MERV, murine endogenous retrovirus.

Antiviral factor		Relation to RTEs	Ligand or target	Antiviral activity	
Gene	Protein			RTEs	Exogenous viruses
*Fv4* (mouse)	Fv4	eMLV Env-derived	mCAT-1	eMLV	eMLV
*Rmcf* (mouse)	Rmcf	MCF virus Env-derived	XPR1	Nonecotropic MLV	Nonecotropic MLV
*Refrex1* (cat)	Refrex-1	ERV-DC Env-derived	COPT1	ERV-DC	ERV-DC
*Felix* (cat)	FeLIX	FeLV-B Env-derived	PiT1	FeLV-B	FeLV-B
*ERVS71-1* (human)	ENVT1	HERV-T Env-derived	MCT-1	HERV-T (defective)	HERV-T (extinct)
ERVH48-1 (human)	Suppressyn	ERVH48-1 Env-derived	ASCT2	ERVH48-1 (defective)	RD114/mammalian type-D retroviruses
*BST2* (human)	BST2; Tetherin	Antagonized by HERV-K(HML-2) Env	Env, budding	ERVs, Exosomes, LINEs	Retroviruses, Enveloped Viruses
*Fv1* (mouse)	Fv1	MERV-L Gag-derived	Capsid uncoating	MLV	MLV, diverse retroviruses
*enJS56A1* (sheep)	enJS56A1	JSRV Gag-derived	Capsid assembly	enJSRV	JSRV
Apobec3 (mouse)	ABEC3	MLV insertion in the *Rfv3* allele	DNA, RNA	ERVs, LINEs	MLV
*APOBEC3B* (human)	ABC3B	Regulation by nearby HERV-H	DNA, RNA	ERVs, LINEs	Retroviruses, Hepadnaviruses
*AIM2* (human)	AIM2	IFN inducibility by MER41	Cytoplasmic DNA	Untested	Diverse DNA and RNA viruses
*IFI6* (human)	IFI6	IFN inducibility by MER41	Unclear	Untested	Diverse DNA and RNA viruses
*GBP2* (human)	GBP2	Driven by ERV9 LTR12	Env processing	HERV-K(HML-2) pseudotypes	Diverse enveloped viruses
*GBP5* (human)	GBP5	Driven by ERV9 LTR12	Env processing	HERV-K(HML-2) pseudotypes	Diverse enveloped viruses
*TLR7* (human)	TLR7	Regulation by nearby HERV-H	Endosomal ssRNA	ERVs, LINEs, SINEs	Diverse ssRNA viruses
*TLR8* (human)	TLR8	Regulation by nearby HERV-H	Endosomal ssRNA	ERVs, LINEs, SINEs	Diverse ssRNA viruses

## Teaser

Mammalian hosts continue to sense, control and even enlist endogenous retroelements in the evolution of antiviral defenses
